# MGPPI: multiscale graph neural networks for explainable protein–protein interaction prediction

**DOI:** 10.3389/fgene.2024.1440448

**Published:** 2024-07-15

**Authors:** Shiwei Zhao, Zhenyu Cui, Gonglei Zhang, Yanlong Gong, Lingtao Su

**Affiliations:** College of Computer Science and Engineering, Shandong University of Science and Technology, Qingdao, China

**Keywords:** PPI prediction, multiscale GNN, model interpretability, key binding residues, Grad-WAM

## Abstract

Protein-Protein Interactions (PPIs) involves in various biological processes, which are of significant importance in cancer diagnosis and drug development. Computational based PPI prediction methods are more preferred due to their low cost and high accuracy. However, existing protein structure based methods are insufficient in the extraction of protein structural information. Furthermore, most methods are less interpretable, which hinder their practical application in the biomedical field. In this paper, we propose MGPPI, which is a Multiscale graph convolutional neural network model for PPI prediction. By incorporating multiscale module into the Graph Neural Network (GNN) and constructing multi convolutional layers, MGPPI can effectively capture both local and global protein structure information. For model interpretability, we introduce a novel visual explanation method named Gradient Weighted interaction Activation Mapping (Grad-WAM), which can highlight key binding residue sites. We evaluate the performance of MGPPI by comparing with state-of-the-arts methods on various datasets. Results shows that MGPPI outperforms other methods significantly and exhibits strong generalization capabilities on the multi-species dataset. As a practical case study, we predicted the binding affinity between the spike (S) protein of SARS-COV-2 and the human ACE2 receptor protein, and successfully identified key binding sites with known binding functions. Key binding sites mutation in PPIs can affect cancer patient survival statues. Therefore, we further verified Grad-WAM highlighted residue sites in separating patients survival groups in several different cancer type datasets. According to our results, some of the highlighted residues can be used as biomarkers in predicting patients survival probability. All these results together demonstrate the high accuracy and practical application value of MGPPI. Our method not only addresses the limitations of existing approaches but also can assists researchers in identifying crucial drug targets and help guide personalized cancer treatment.

## 1 Introduction

PPIs are the basic components of protein complexes, which play crucial roles in cellular components and biological processes (Chen and Zacharias, 2023; Wu et al., 2023; Reed et al., 2024). Dysfunction interactions can lead to various chronic diseases and even cancer. Moreover, PPI contains a wealth of information, such as receptor binding and immune response, and can providing key insights of protein functionality and potential therapeutic targets for human cancer (Kjer-Hansen and Weatheritt, 2023; Rodina et al., 2023). As a results, numerous computational methods have been developed for PPI prediction, which are more efficient and low cost in comparison with traditional experimental based methods.

Early computational methods primarily use protein sequence as input (Ding and Kihara, 2018; Wang et al., 2021; Boldridge et al., 2023; Gainza et al., 2023; Shen et al., 2023). Such methods based on the hypothesis that proteins with similar sequences tend to have similar binding tendencies. With the increasing availability of protein structure data, especially with the emergence of Alphafold2 (Bryant et al., 2022), structure-based PPI prediction algorithms (Dong et al., 2019; Tang et al., 2023) have been growing year by year. In comparison with sequence-based methods, structure-based methods can capture more detailed binding structure information, therefore, the prediction accuracy are often more higher. PPI prediction methods can be categorized into two groups: machine learning and deep learning based methods. Among them, Support Vector Machines (SVM) (Guo et al., 2008; Wong et al., 2015; Bandyopadhyay and Mallick, 2016; Zhou et al., 2017) based methods aim to find an optimal hyperplane using protein sequence information (Chen and Jeong, 2009; Xia et al., 2010; Zahiri et al., 2013; You et al., 2015), 3D structure (Li et al., 2012), and domain information (Chen and Liu, 2005) to maximize the margin between different proteins for classification. Decision tree-based methods, on the other hand, utilize features such as protein 3D structure, primary sequence, and domain composition for PPI prediction. Compared to traditional machine learning-based methods, deep learning has the ability to automatically learn higher-level feature representations. Among them, DeepFE-PPI (Yao et al., 2019) proposes a novel residue representation method and deep learning network for protein-protein interaction prediction. Deep-Trio (Hu et al., 2022) introduces a sequence-based approach for PPI prediction, utilizing multiple parallel convolutional neural networks. GNN-PPI (Jha et al., 2022) leverages graph neural networks and language models (LM) to extract high-quality features from proteins for predicting protein interactions. HIGH-PPI (Gao et al., 2023) consists of bottom-level protein graph neural network (BGNN) representation learning and top-level PPI graph neural network (TGNN) representation learning. The vector representations obtained from both networks are concatenated to obtain the final prediction result.

While existing methods have achieved promising results on datasets from various species, most of them lack sufficient protein feature extraction and interpretability. Sequence-based models primarily focus on the one-dimensional sequence characteristics of proteins, while neglecting the higher-order structural properties. This can lead to incomplete accuracy in predicting PPIs since structural information plays a crucial role. Even when some models take into account the structural information of proteins, they often fail to adequately address how to extract both global and local structural information from proteins to contribute to PPI prediction. Additionally, some models incorporating interpretable modules solely rely on spatial biological arrangements of residues, introducing uncertainties and challenges to scientific validity and reliability. These limitations hinder their practical applications in PPI prediction. To address the issue of insufficient protein feature extraction, we choose to utilize graph convolutional layers to capture as much global structural information of protein graphs as possible. However, we need to find a suitable trade-off due to challenges such as over smoothing and gradient vanishing, which may arise when using multiple graph convolutional layers. Secondly, given the uniqueness of proteins, GNN should preserve the local structural information of proteins. Certain amino acid residues are crucial for protein interactions, and even the presence of specific residues determines protein functionality. Therefore, GNN should effectively distinguish between important residues and less relevant ones, enabling reasonable judgments in subsequent site prediction experiments. Secondly, the current interpretability of PPI models based on graph neural networks falls short in translating interactions into an understanding of function and mechanism. Moreover, explanations based solely on spatial biological arrangement information of residues are insufficient. Sequence information provides only static insights, disregarding the importance of protein structure and dynamic characteristics in determining the occurrence and stability of interactions.

To address the limitations in protein feature extraction and interpretability, we propose a novel framework called MGPPI, our main contributions are as follows.

• We represent both interacting proteins as amino acid level graphs, with amino acids as nodes and various relationships between them as edges, which allows MGPPI to capture the internal structure of proteins and their interactions more accurately.
• To address the issue of black-box features in existing deep learning models, we propose a novel interpretability module called Grad-WAM. Grad-WAM utilizes the gradient magnitudes generated by the final Graph Convolutional Network (GCN) layer of the model to calculate the contributions of each amino acid position in the PPI prediction. This information is then used to visualize the crucial amino acid residues that play a key role in the interaction between the two proteins.
• To address the issue of insufficient protein structure feature extraction in existing models, we propose a Multiscale Graph Convolutional Neural Networkk (MGCN) to learn both local and global protein structural representations. These representations are mapped into feature vectors for each protein, and the are combined for PPI prediction.

## 2 Methods

### 2.1 Input representation

**FIGURE 1:**
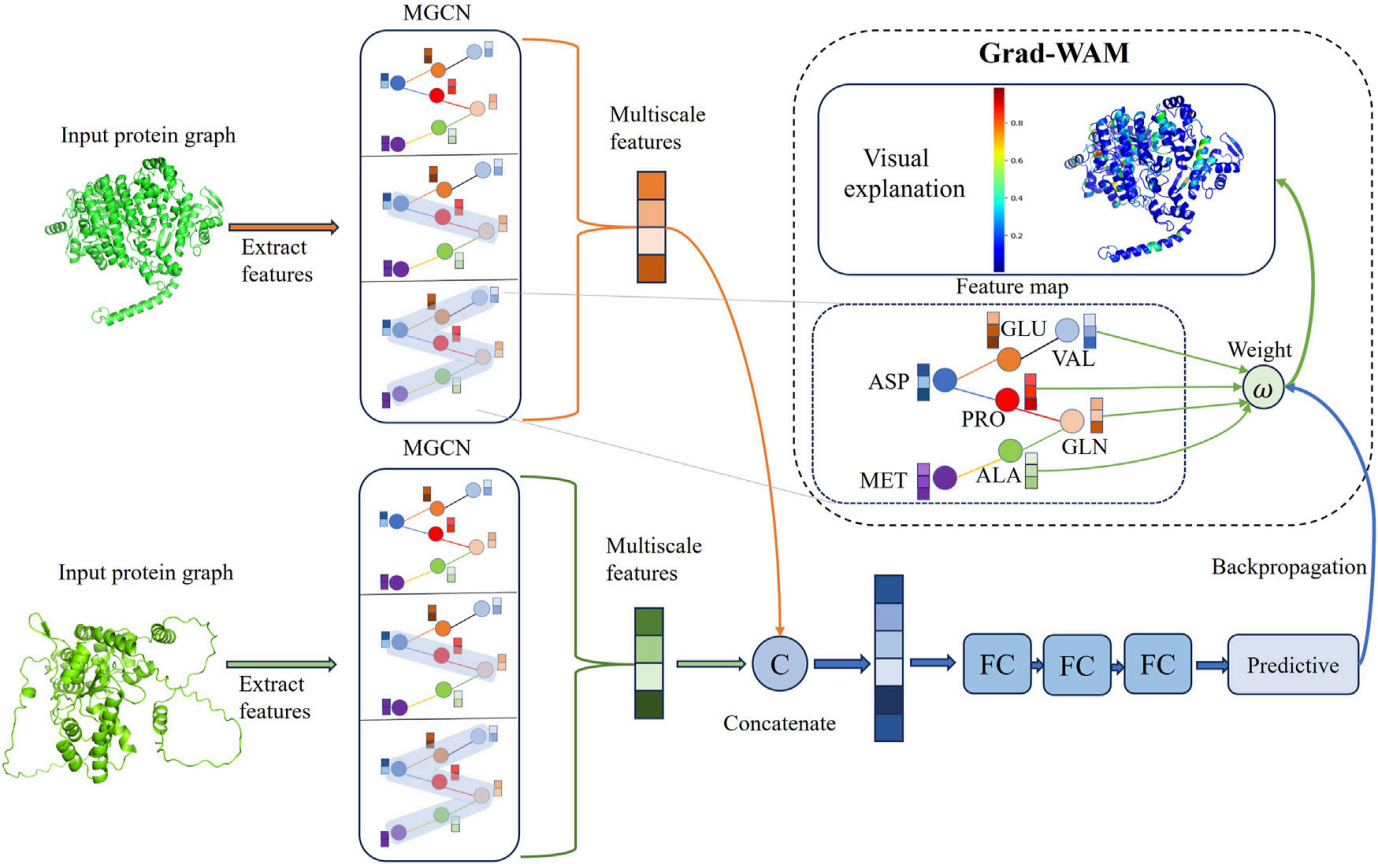
The model takes protein structures as input and employs graph representation learning to feed them into MGCN for extracting multi-scale features of proteins, resulting in the final protein representation vectors. The two protein representation vectors are then fused to obtain a combined representation of the protein pair. Subsequently, the combined representation is fed into fully connected layers to output predicted interaction scores. GradWAM utilizes the gradient information from the last graph convolutional layer of MGCN and the final predicted scores to analyze the importance of each amino acid for protein-protein interactions.

The workflow of MGPPI is shown in Figure 1. The input to the model is paired protein structures, which are represented as amino acid level graphs G = (N, E). Where nodes (N) are amino acid residues (i.e., amino acid-level graph representation) and the relationships between amino acids as edges (E). Node attributes including solvent-accessible surface area, ϕ angle, ψ angle, secondary structure (alpha helix, isolated beta-bridge residue, strand, 3–10 helix, turn, bend), AAPHY7 and BLOSUM62 descriptors, hydrogen bond acceptor and donor information. Edge attributes including information about the existence of covalent bond, hydrophobic contact, ionic bond, disulfide bond, hydrogen bond, and aromatic bond relationships between amino acids as edge attributes. The details are shown in Table 1 below.

**TABLE 1:** Protein graph representation at the amino acid level.

Name	Size
Node features	
BLOSUM62 descriptors	23
AAPHY7 descriptors	7
One-hot encoded belonging to secondary structure	6
Solvent-accessible surface area	1
ϕ angle (divided by 180)	1
ψ angle (divided by 180)	1
Hydrogen bond acceptor	1
Hydrogen bond donor	1
Edge features	
Covalent bond	1
Hydrophobic contact	1
Hydrogen bond	1
Aromatic bond	1
Ionic bond	1
Disulfide bond	1

### 2.2 Graph neural network

**FIGURE 2:**
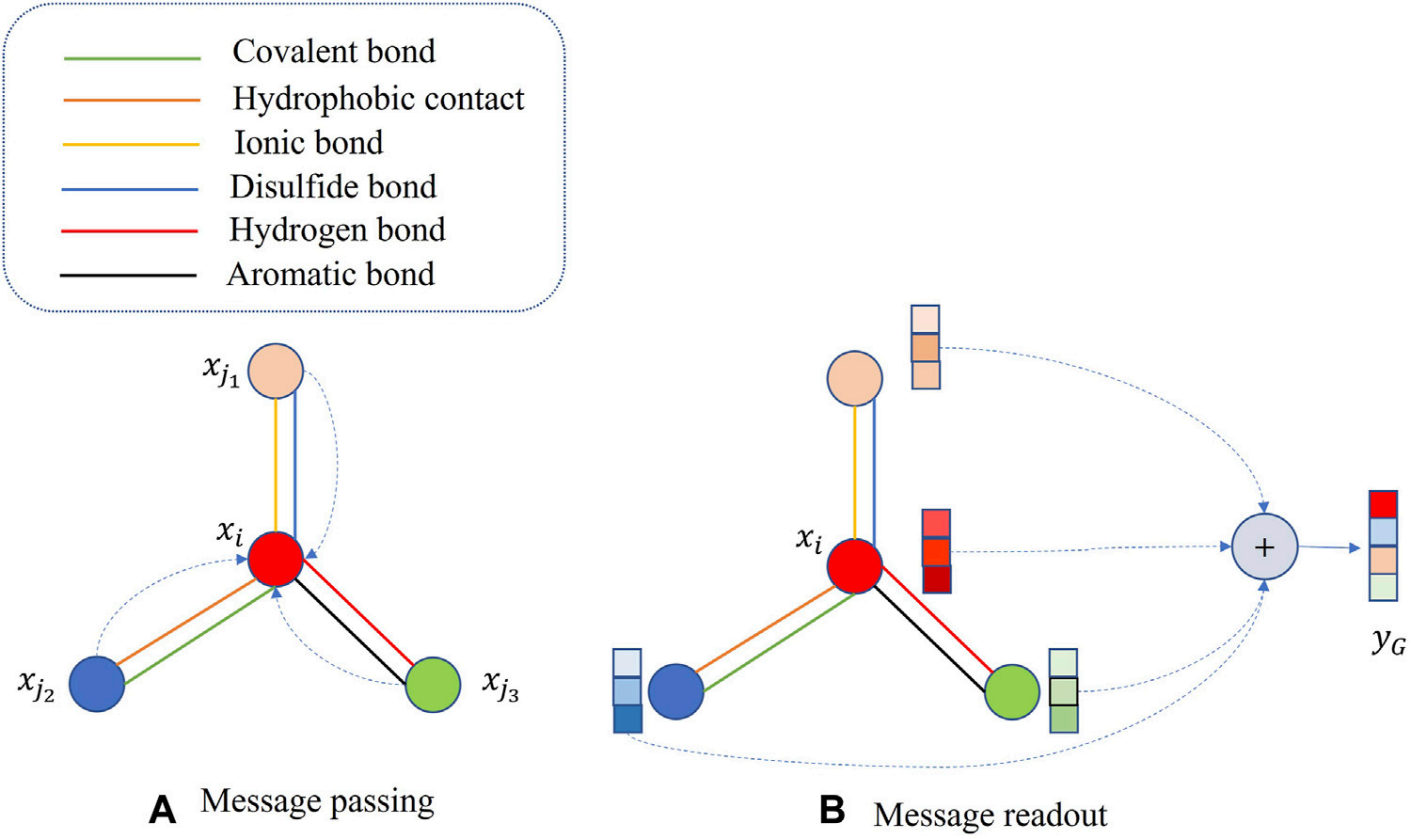
Protein graph representation learning. **(A)** message passing phase, **(B) **message readout phase.

We extract protein features using Graph Neural Networks and combine the feature vectors of two proteins to predict Protein-Protein Interactions. We map the protein graph representation to feature vectors through two stages: message passing and readout. In the message passing stage, as shown in Eq. 1, corresponding to Figure 2A, we update the feature vector of each node by incorporating the feature information from its neighboring nodes.

$$x_{i}^{\left(T\right)}=\sigma \left(\Phi_{1}x_{i}^{\left(T-1\right)}+\Phi_{2}\underset{j\in N\left(i\right)}{\sum }x_{j}^{\left(T-1\right)}\right)$$ (1)

$x_{i}^{\left(T\right)}$ represents the feature vector of the *i*th node at a time step T; $\Phi_{1},\Phi_{2}$ symbolize learnable weight matrices shared by all nodes, initialized with small random values pre-training, and continuously optimized during training; σ encompasses two operations: node-level batch normalization (Li et al., 2021) and the ReLU activation function; N(i) refers to the set of neighboring nodes for the *i*th node. By utilizing Eq. 1, nodes can gradually capture more global information from the protein graph representation.

$$y_{G}=\frac{1}{\left|M\right|}\underset{i\in M}{\sum }{x_{i}}^{\left(T\right)}$$ (2)

M represents the set of all amino acids in the protein. During the readout stage, as shown in Eq. 2, corresponding to Figure 2B, we obtain the representation vector, denoted as $y_{G}$ , for the entire protein graph G .

### 2.3 Multiscale graph convolutional neural network for protein encoding

**FIGURE 3:**
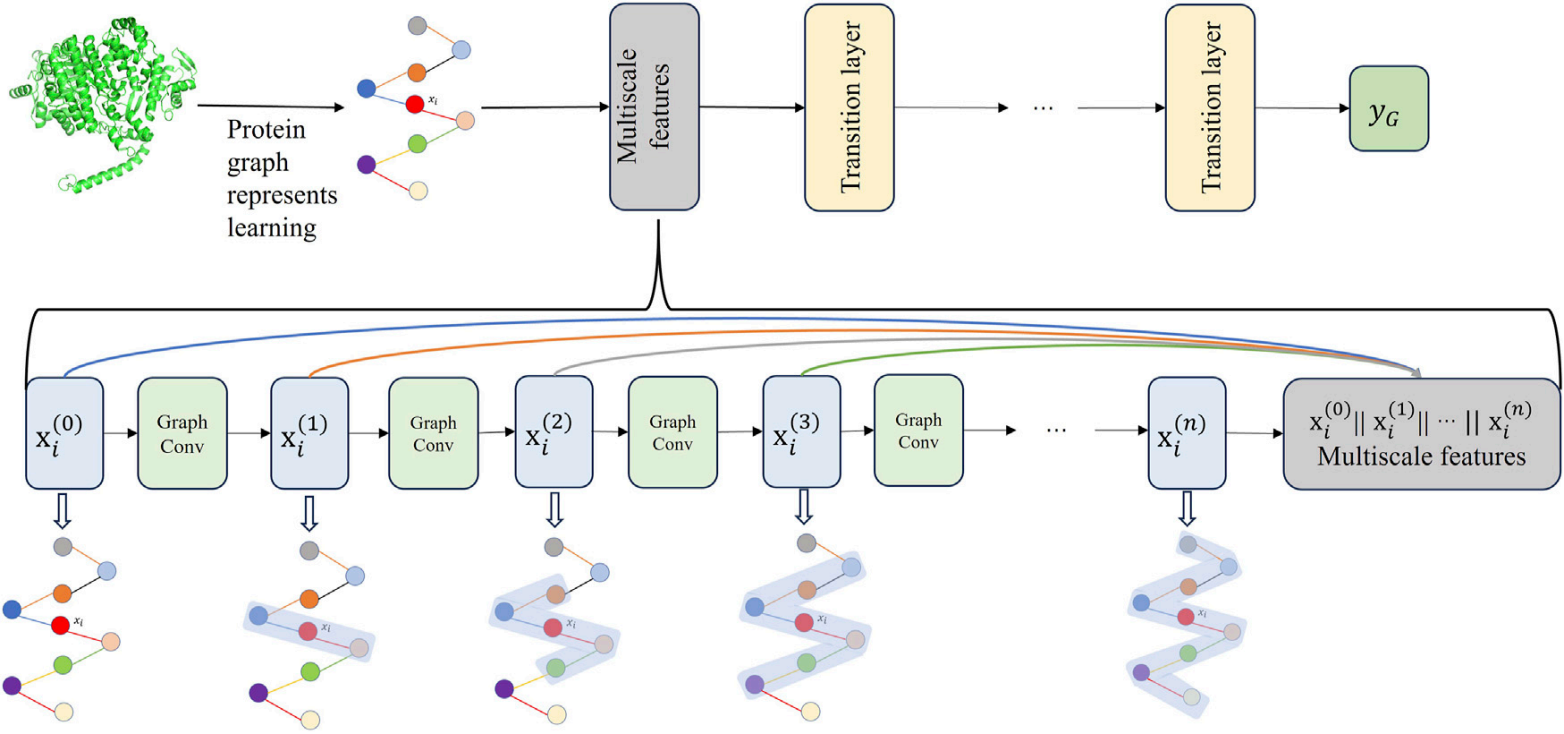
The upper part of the figure illustrates the workflow of MGCN, which consists of three multi-scale blocks and three transition layers. The lower part of the figure provides a detailed description of the multiscale blocks.

Graph neural network extract features of target proteins through a layer-wise sampling approach. The layer-wise sampling approach allows the model to extract the node features after each convolutional layer, enabling the model to capture as much of the protein’s global and local features as possible. A small receptive field allows nodes to observe only local protein structures, failing to capture global structural features. As a result, nodes fail to establish connections with the overall protein structure. On the contrary, when the receptive field is too large, nodes may absorb more irrelevant features that are unrelated to protein interactions. Additionally, it can lead to the homogenization of node features within a particular region, giving rise to the problem of oversmoothing. To effectively learn and integrate features from protein graph data at different receptive fields and granularities, we propose a Multiscale graph convolutional neural network (MGCN). MGCN consists of three multiscale blocks and three transition layers, the multiscale blocks as shown in Figure 3, the transition layer transfers the multiscale information of nodes to the next stage. At time step n+1 , the transition layer as shown in Eq. 3.

$$\begin{array}{c} x_{i}^{\left(n+1\right)}=\sigma \left(\Phi_{1}\left(x_{i}^{\left(0\right)}\|x_{i}^{\left(1\right)}\|\cdots \|x_{i}^{\left(n\right)}\right)\right.\\ \left.+\Phi_{2}\underset{j\in N\left(i\right)}{\sum }\left(x_{j}^{\left(0\right)}\|x_{j}^{\left(1\right)}\|\cdots \|x_{j}^{\left(n\right)}\right)\right)) \end{array}$$ (3)

The purpose of the transition layers is to connect adjacent multiscale blocks, facilitating an increase in the depth of MGCN. The multiscale blocks allow gradients to propagate through skip connections, alleviating the issue of gradient vanishing. Additionally, MGCN enhances the representation capability of nodes by concatenating combinations of features from different receptive fields. After passing through the final transition layer, the feature vectors of amino acids are propagated to the readout stage. In the information readout stage, the feature vectors of all amino acids in a protein are integrated and transformed into a single feature vector representing that protein. This protein feature vector is then utilized for subsequent stages of protein-protein interaction prediction.

### 2.4 MGPPI network architecture

After obtaining the vector representations of proteins, we concatenate the vector representations of both proteins and feed them into fully connected layers to predict interaction scores. Since the predicted interaction scores fall within the range of 0–1, we need to set a threshold for classification. As we are going to predict key sites of protein interaction later, we need to minimize the false positive rate as much as possible to enhance the credibility of prediction results, and hence, we have set the classification threshold to 0.7. If the score is greater than this threshold, it is assigned a value of 1, indicating that the two proteins are likely to interact with each other. Conversely, if the score is below the threshold, it is assigned a value of 0, suggesting that the two proteins are less likely to interact or are unable to interact. Each fully connected layer is followed by a ReLU activation function and a dropout layer with a dropout rate of 0.1, consistent with previous studies. There are a total of three fully connected layers. Our loss function uses cross-entropy loss (Yang et al., 2021), defined as follows Eq. 4:

$$CrossEntropyLoss=-\overset{n}{\underset{i=1}{\sum }}Y_{i}\log P_{i}$$ (4)

$P_{i}$ represents the predicted interaction score for the *i*th protein pair, $Y_{i}$ represents the correct interaction score for the *i*th protein pair, and n represents the total number of protein pairs.

### 2.5 Gradient weighted interaction activation mapping

To enhance the interpretability of the model, we propose an interpretable module called Gradient weighted interaction activation mapping (Grad-WAM) and visualize the results. Grad-WAM improves the identification of key interacting amino acid residues and explains the mechanism of protein-protein interactions, effectively addressing the issue of lack of interpretability in neural network predictions. Grad-WAM utilizes the gradient magnitudes generated by the last layer of graph convolutions and the final predicted scores to calculate the contributions of different amino acid positions in the protein structure to protein-protein interaction predictions.

Specifically, Grad-WAM uses a weighted combination of the positive partial derivatives of the feature maps with respect to the interaction values to generate the corresponding visual explanations. Since the contributions of each element are not equal, an additional weight is introduced to weight the gradient values. The calculation formula is as follows:

$$\omega =\underset{i}{\sum }\alpha_{i}\cdot ReLU\left(\frac{\partial P}{\partial T_{i}}\right),\;\forall \left\{i|i\epsilon T\right\}$$ (5)

Where ω represents the weight, and positive gradient values indicate a positive influence on the predicted values, ensuring that ω is a weighted average rather than a global average. $\alpha_{i}$ corresponds to the gradient weight of the *i*th node. ReLU() denotes the ReLU activation function. $T_{i}$ is the feature value of the *i*th node in the feature map T of the last graph convolutional layer. P represents the predicted protein-protein interaction value, and the calculation formula is as follows:

$$P=\underset{i}{\sum }\alpha_{i}\cdot ReLU\left(\frac{\partial P}{\partial T_{i}}\right)\cdot T_{i}$$ (6)

The derivative of Eq. 6 with respect to the variable yields the following Eq. 7:

$$\frac{\partial P}{\partial T_{i}}=\alpha_{i}\cdot \frac{\partial P}{\partial T_{i}}+T_{i}\cdot \alpha_{i}\cdot \frac{\partial^{2}P}{{\left(\partial T_{i}\right)}^{2}}$$ (7)

Rearranging the terms in Eq. 7 yields the following Eq. 8:

$$\alpha_{i}=\frac{\frac{\partial P}{\partial T_{i}}}{\frac{\partial P}{\partial T_{i}}+T_{i}\cdot \frac{\partial^{2}P}{{\left(\partial T_{i}\right)}^{2}}}$$ (8)

Substituting the weight $\alpha_{i}$ from Eq. 8 into Eq. 5, yields the final weight as shown in Eq. 9:

$$\omega =\underset{i}{\sum }\left[\frac{\frac{\partial P}{\partial T_{i}}}{\frac{\partial P}{\partial T_{i}}+T_{i}\cdot \frac{\partial^{2}P}{{\left(\partial T_{i}\right)}^{2}}}\right]\cdot \;ReLU\left(\frac{\partial P}{\partial T_{i}}\right),\forall \left\{i|i\epsilon T\right\}$$ (9)

The contribution of different amino acids at various positions in the protein structure to the prediction of protein-protein interactions can be calculated using Eq. 9. A key amino acid that plays a crucial role in protein-protein interactions is annotated and displayed in the protein structure. The color gradient from blue to green to red represents the contribution values of the amino acids, with higher contribution values indicated by a redder color, indicating their significant role in protein interactions. This method enhances the interpretability of the model. Grad-WAM calculates the contribution of different amino acid positions in protein-protein interaction prediction within protein structures by utilizing the gradient magnitude generated by the final graph convolutional layer and the predicted values propagated through backpropagation. Due to the local connectivity and weight sharing structure employed by the graph convolutional layer, it preserves spatial information lost in the fully connected layers. The last graph convolutional layer strikes a balance between high-order semantics and detailed spatial information (Selvaraju et al., 2017), considering both global and local features. Finally, the minimum-maximum normalization method is used to map the impact probabilities of each amino acid on protein interactions, ranging from 0 to 1.

### 2.6 Data

The datasets used in this study include the Human Protein Reference Database (HPRD) (Peri et al., 2003), the Online Predicted Human Interaction Database (OPHID) (Brown and Jurisica, 2005), the H. sapiens dataset from the Biological General Repository for Interaction Datasets (BioGRID) (Oughtred et al., 2019), and the STRING database (Szklarczyk et al., 2019). Additionally, the negative samples in the HPRD dataset are sourced from curated negative protein-protein interaction datasets. The negative protein-protein interaction datasets collected data on human protein pairs that did not exhibit interactions in large-scale yeast two-hybrid screening. The quantities of positive and negative samples after processing for each dataset are shown in Table 2.

**TABLE 2:** The quantity of positive and negative samples in each dataset.

Dataset	Positive	Negative
HPRD	35944	763115
OPHID	39412	63932
BioGRID	72367	82731
STRING	11810480	167224
Multi-species	25640	30332

For the aforementioned datasets, protein names were converted to UniProt (Bateman, 2019) ID, and the corresponding PDB (Berman et al., 2000) files were collected for training, testing, and validation purposes. We randomly sampled 25,000 positive examples and 25,000 negative examples from each human protein dataset, resulting in a final training set of 200,000 samples. The training set is independent of the subsequent test set.

### 2.7 Experimental environment configuration

The experimental environment consisted of Ubuntu 20.04.6LTS, an Intel (R) Core (TM) i5-10400 CPU, and an NVIDIA Corporation GP102G (Tesla P40) GPU. A batch size of 512 was set, and the Adam optimizer with a learning rate of 0.0005 was used to update the model parameters. The MGCN architecture comprised 15 graph convolutional layers, including three multiscale blocks. Each multiscale block consisted of N (N = 4) graph convolutional layers and three transition layers.

## 3 Result and discussion

### 3.1 Compare on human proteins datasets

**FIGURE 4:**
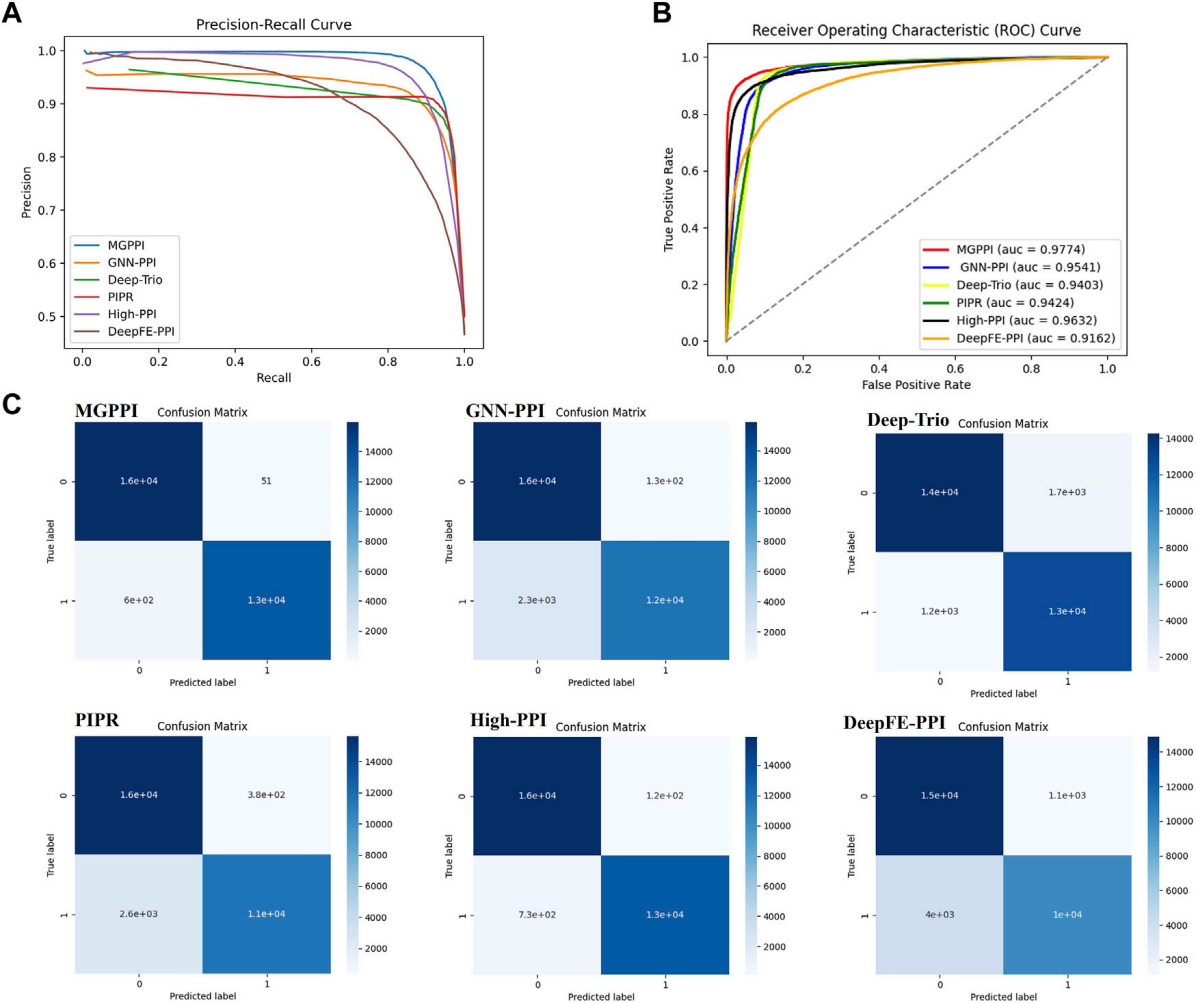
Here are the precision-recall curves **(A)**, ROC curves **(B)**, and AUC for the six methods on the HPRD dataset.**(C)** represents the 20% test set selected from the BioGRID dataset for evaluating MGPPI and five other methods. The confusion matrix was obtained by applying a threshold of 0.7 to the predicted values (as the predictions are continuous values between 0 and 1).

We selected the HPRD as the benchmark dataset and compared MGPPI with several state-of-the-art PPI prediction methods for analysis. These methods include High-PPI, a hierarchical graph neural network-based PPI prediction method; GNN-PPI, a method that utilizes graph neural networks to learn PPI network topological structures; Deep-Trio, a deep learning framework based on a masked multiscale CNN architecture that learns multiscale contextual information from protein sequences; PIPR (Chen et al., 2019), an end-to-end framework based on recursive neural networks (RNNs) that incorporates pre-trained residue embeddings for protein representation; and DeepFE-PPI, a method that employs residue representation using the Res2vec [based on Word2vec (Mikolov et al., 2013)] approach. In Figure 4A, the precision-recall curves are provided, while Figure 4B presents the ROC curves. Across both evaluation metrics, MGPPI consistently achieved the best performance among all the compared methods, this also highlights the significance of protein structural information in PPI prediction. Furthermore, we conducted testing on the BioGRID dataset for MGPPI and five other methods, resulting in the confusion matrix shown in Figure 4C. The conclusions remain consistent with the previous findings.

**FIGURE 5:**
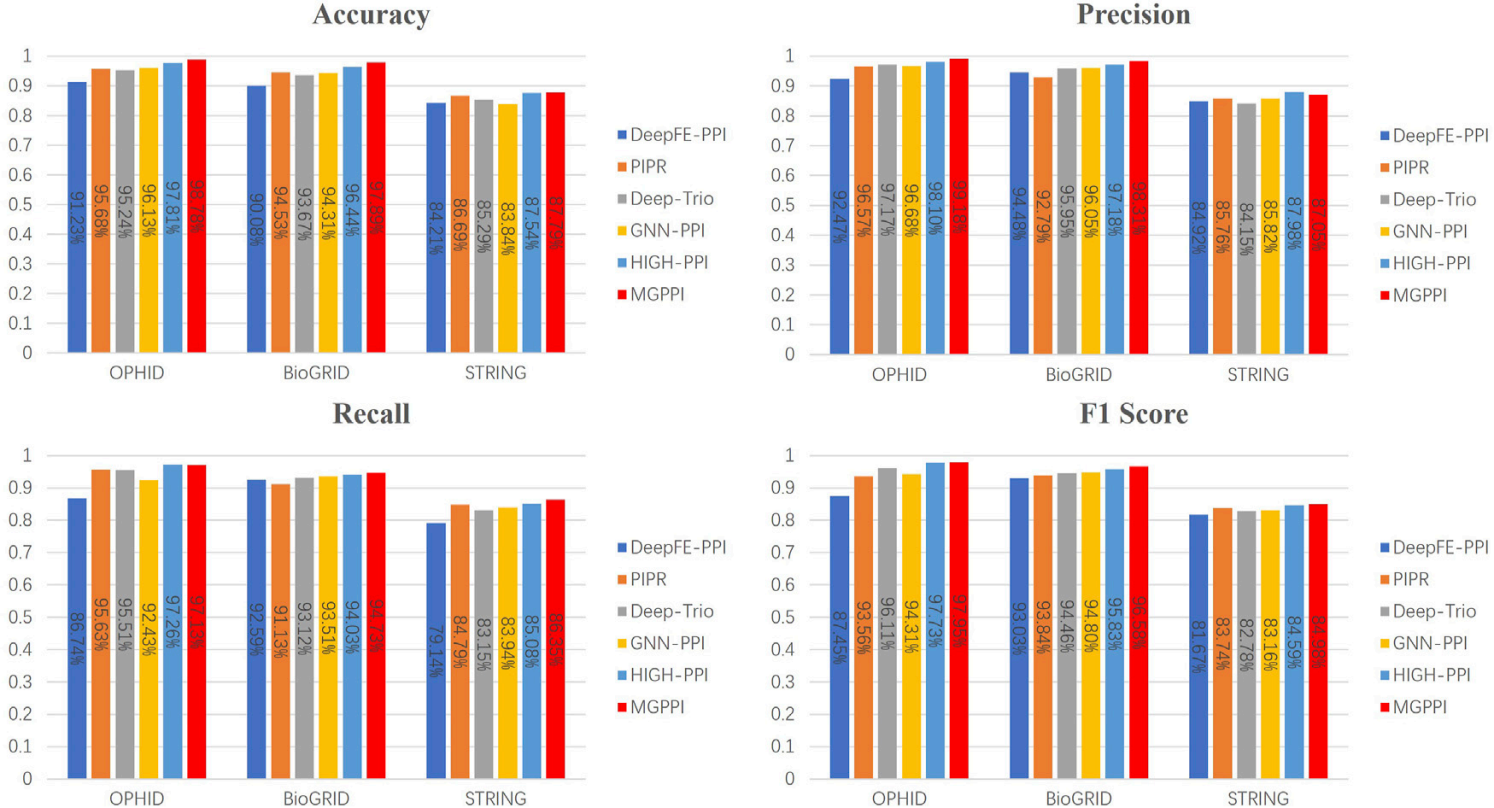
The overall performance of MGPPI compared to five other PPI prediction methods was evaluated on the OPHID dataset, the *H. sapiens* dataset from BioGRID, and STRING.

To further validate the predictive capability of MGPPI for protein-protein interactions, we conducted performance comparisons between MGPPI and the five different PPI prediction methods on three other human protein datasets. The results as shown in Figure 5.

From Figure 4 and Figure 5, it can be observed that our proposed method, MGPPI, demonstrates favorable performance across various commonly used evaluation metrics. MGPPI aims to comprehensively consider the impact of oversmoothing and gradient vanishing while extracting as much global information as possible from protein graph structures. It simultaneously takes into account the preservation of local information within protein graph structures to enhance the prediction performance of PPI. On the other hand, High-PPI effectively utilizes a layered modeling approach. In this approach, the inner layer of the protein view consists of residues as nodes, with their physical adjacency forming the edges. The outer layer of the protein view considers proteins and their interactions as nodes and edges, respectively, in the PPI network structure. High-PPI, ranking second, highlights the significance of protein structural information in PPI prediction. In comparison to other sequence-based prediction methods such as DeepFE-PPI, PIPR, and Deep-Trio, MGPPI exhibits significant advantages, further highlighting the importance of protein structure information in PPI prediction.

### 3.2 Compare on multi-species proteins datasets

The multi-species dataset consists of three species: *Caenorhabditis elegans* (Celegan), *Drosophila melanogaster* (*Drosophila*), and *Escherichia coli* (Ecoli). The utilization of multi-species datasets enables further exploration of the practical and generalization capabilities of MGPPI. Experimental evaluations conducted on these datasets assess the model’s ability to generalize, as all previous models were trained and tested solely on human datasets. For MGPPI, protein network construction necessitates corresponding PDB files, obviating the necessity to establish distinct thresholds based on sequence similarity for data categorization. We standardized the dataset into a comprehensive multi-species dataset to assess the performance of each model effectively. The results as shown in Table 3.

**TABLE 3:** Comparison of MGPPI with other methods on a multi-species dataset (%).

Method	Accuracy	Precision	Recall	F1
DeepFE-PPI	68.70	63.97	66.53	65.22
PIPR	73.92	65.98	73.15	68.69
Deep-Trio	71.29	69.57	70.67	68.54
GNN-PPI	67.15	61.53	64.82	62.10
High-PPI	75.18	73.26	75.21	73.52
MGPPI	**82.53**	**81.77**	**83.68**	**82.17**

Based on Table 3, it can be observed that MGPPI exhibits remarkable generalization ability. This can be attributed to its capability of performing graph representation learning on proteins from different species. MGPPI effectively extracts valuable structural information, enabling accurate prediction of protein-protein interactions. On one hand, sequence-based methods can accurately predict some PPI by identifying similarities between amino acid sequences of non-human species and those of human proteins, inferring similar functionalities and interaction tendencies. However, it should be noted that not all proteins from other species can be matched with similar sequences to human proteins. On the other hand, GNN-PPI might not have encountered multi-species data and thus struggles to accurately construct the PPI network structure, this leads to a performance deviation of the model from the anticipated expectations.

### 3.3 Ablation study

As the depth of GNN models increases, we have observed the issue of over-smoothing in certain cases. Over-smoothing refers to a situation where nodes incorporate an increasing amount of information from their neighboring nodes, causing the representation vectors of some nodes to converge towards the same value. When most or all nodes have representation vectors that converge to the same or a few values, it hinders the normal learning process of the model and renders the neural network’s output insensitive to the input information. Therefore, in this paper, we propose MGCN to address this problem by integrating representation vectors from different temporal nodes, preserving a combination of receptive fields at different scales. This approach enhances the model’s ability to represent nodes and alleviates the issue of over-smoothing.

In MGPPI, we alleviate the issues of over-smoothing and gradient vanishing by leveraging a multiscale module and batch normalization techniques to improve model performance. To demonstrate the individual contributions of the multiscale module and batch normalization, We conducted ablation study on the HPRD dataset. The study consisted of three experimental scenarios:

(1) The first scenario involved removing batch normalization while retaining the multi-scale module.

(2) The second scenario involved removing the multiscale module and utilizing the four graph convolution layers without it.

(3) The third scenario retained both the multiscale module and batch normalization for experimental analysis.

The results presented in Table 4 indicate that both the multi-scale module and batch normalization are essential components of MGCN. Furthermore, experiments conducted on the HPRD dataset aimed to investigate the impact of receptive field on the model’s performance. Specifically, we progressively increased the number of graph convolutional layers (i.e., 2, 3, 4, 5, 6) within the multi-scale module to enlarge the network’s receptive field.

**TABLE 4:** Investigating the individual contributions of the multiscale module and batch normalization (%).

Model	Loss	Accuracy	Precision	Recall	F1	MCC
Without multiscale module	1.50	98.16	99.31	95.86	97.53	95.07
Without batch normalization	1.34	98.28	99.29	95.61	97.45	95.15
**MGPPI**	**1.16**	**98.74**	**99.52**	**96.11**	**97.78**	**95.41**

From the results shown in Table 5, it can be observed that, overall, increasing the number of convolutional layers improves the overall performance of the model. However, when using six convolutional layers, some metrics are not as good as those achieved with four or five layers. This discrepancy may arise due to the inclusion of noise information from residues that do not participate in protein-protein interactions when increasing the number of convolutional layers. Considering that each additional convolutional layer introduces more computational operations, which could result in longer training and inference times, we aimed to strike a balance between the model’s overall performance and its time complexity. Furthermore, we aimed to mitigate the impact of over-smoothing issues and gradient vanishing issues. Therefore, after comprehensive consideration, we opted for a compromise solution, retaining four convolutional layers for subsequent experiments.

**TABLE 5:** Investigating the impact of different numbers of convolutional layers within the multiscale module on the model (%).

Convolutional layer	Loss	Accuracy	Precision	Recall	F1	MCC
2	1.97	97.87	98.86	95.33	96.86	94.78
3	1.54	98.04	99.14	95.72	97.18	95.09
4	1.16	**98.74**	99.52	**96.11**	**97.78**	95.41
5	**1.12**	98.63	**99.56**	96.11	97.64	95.35
6	1.29	98.40	99.39	95.93	97.43	**95.46**

### 3.4 Predict and validate binding sites

**FIGURE 6:**
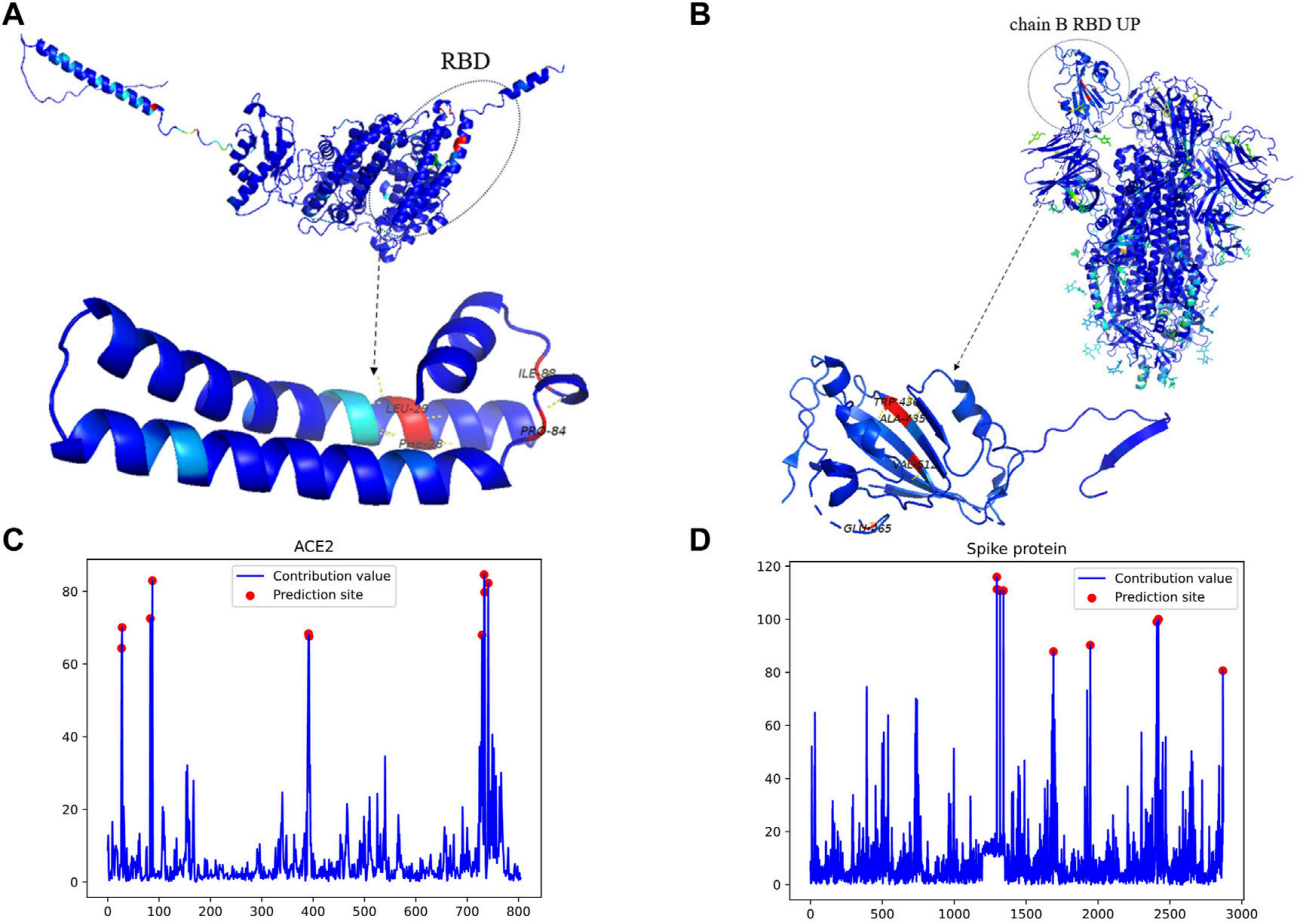
The key binding sites and amino acid contributions of the Spike protein and the human receptor ACE2. **(A)** represents the visualization results and predicted binding sites for the human receptor protein ACE2. **(B)** Represents the visualization results and predicted binding sites for the Spike protein. **(C)** Displays the contribution values of all amino acids in ACE2, represented by a single amino acid chain. **(D)** Displays the contribution values of all amino acids in the Spike protein, which consists of three amino acid chains: **(A, B, and C)**. Furthermore, the receptor binding domains on chains A and C are oriented downward, while the receptor binding domain on chain B is oriented upward. In the visualization, the region to the left of position 1,500 with higher scores corresponds precisely to the amino acids 435Ala, 436Trp, 512Val, and 465Glu in the B chain. Since the calculated contribution values are relatively small, we have proportionally magnified them for better color distinction during the visualization stage.

To validate the interpretability and binding site prediction capability of MGPPI, we conducted an experiment using the interaction between the spike protein of SARS-COV-2 and the human ACE2 receptor protein as a case study. After feature extraction, graph convolution operations, and visualization, we discovered a high interaction score between the two proteins, indicating their ability to interact, which aligns with existing research findings. Additionally, using Grad-WAM visualization, we identified key amino acid residues that play a crucial role in their binding, elucidating the specific binding sites between Spike protein and Ace2. As show in Figure 6. We uniformly amplified the contribution values of all amino acids by a factor of 100 to facilitate color differentiation of the amino acids during the visualization process, ultimately resulting in the contribution value plots shown in Figures 6C,D.

As shown in Figure 6B, the residues 435Ala, 436Trp, 512Val, and 465Glu located in the receptor binding domain of the Spike protein’s B chain exhibit the highest contribution values, indicating their significant impact in the interaction with the human receptor protein ACE2 (Chi et al., 2020). Have revealed that the receptor binding domains of the other two chains in the Spike protein are oriented downward, while the receptor binding domain on the B chain is oriented upward. This orientation suggests that the receptor binding domain on the B chain, which is typically the first to come into contact with the human receptor protein, further supports the reliability of the predicted binding sites. To further validate our findings, we mapped these predicted binding sites to functional domains and identified their corresponding functional annotations. The identified sites are located within the IPR018548 and IPR042578 functional domains, and the respective functional annotations of these domains are as follows:

The IPR018548 domain functions as the spike protein S1 subunit, receptor binding domain, and β -coronavirus. The IPR042578 domain functions as the spike protein S1, S2, and S2′, where S1 is responsible for binding to host cells and initiating infection, S2 is involved in cell membrane fusion, and S2’ facilitates viral fusion.

The GO annotation (GO:0039654) indicates involvement in the fusion of viral membrane with the host endosomal membrane. The GO annotation (GO:0019064) suggests involvement in the fusion of viral membrane with the host ER membrane. The GO annotation (GO:0016020) indicates participation in the entry of the virus into host cells through endocytosis.

The functional domain and Gene Ontology annotations associated with these predicted binding sites provide additional evidence to substantiate the reliability and scientific validity of our predictions. These functional domains, such as IPR018548 and IPR042578, along with the corresponding GO annotations, further support the significance of the predicted binding sites in terms of their functional relevance and their involvement in critical viral-host interactions.

As shown in Figure 6A, The predicted binding sites, 28Phe, 29Leu, 84Pro, and 88Ile, are located on the edges of ACE2 and are prone to interact with other proteins. These sites can be mapped to specific functional domains, with corresponding Gene Ontology (GO) annotations. However, upon reviewing published literature, the actual binding sites are on the frontward-facing region of the protein, specifically the residues 28Phe, 29Leu, 84Pro, and 88Ile, with the following corresponding Gene Ontology (GO) annotations:

GO:0006508 (Protein catabolic process by peptide bond hydrolysis): This annotation suggests that these binding sites may be involved in the hydrolysis of peptide bonds, leading to the breakdown of larger polypeptides into smaller ones or amino acids. GO:0008237 (Metalloendopeptidase activity): This annotation indicates that the binding sites may possess the enzymatic activity of a metalloendopeptidase, which involves the cleavage of peptide bonds within a protein. GO:0008241 (Peptidyl-dipeptidase activity): This annotation suggests that these sites may catalyze the release of C-terminal dipeptides from peptide chains. GO:0016020 (Membrane and protein complex-associated within lipid bilayer): This annotation implies that the proteins containing these binding sites are embedded within the lipid bilayer and associated with protein complexes.

The functional domain allocation and GO annotation of predicted binding sites provide scientific evidence for the potential roles of these sites in the interaction between ACE2 and spike protein. This further strengthens the reliability of the predicted binding sites. Additionally, the use of Grad-WAM allows for intuitive visualization of the prediction results, enabling researchers to perform more targeted experimental validations based on the obtained amino acid contribution values. Furthermore, through MGPPI and Grad-WAM, we elucidated the roles of the two protein interactions in cellular processes and the functional significance of the predicted sites in the interaction process, thus enhancing the practicality and scientific rigor of MGPPI.

### 3.5 The impact of the predicted mutation site on cancer patients

**FIGURE 7:**
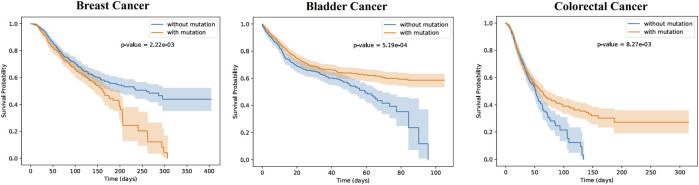
The patients with the three types of cancer were divided into two groups each, based on the occurrence of mutations in the key sites (mutated group) or the absence of mutations (non-mutated group). Survival probability differences over time were analyzed for the two patient groups of each cancer type, as depicted in the figures. The shaded regions around the curves represent the confidence intervals. The *p*-values were found to be 1.57e-03, 5.19e-04, and 8.27e-03, indicating statistically significant differences in survival probabilities between the two patient groups for all three types of cancer. This suggests that the presence of mutations has an impact on patient survival. In the case of breast cancer, the non-mutated group exhibited higher survival probabilities over time compared to the mutated group. Conversely, for the other two types of cancer, the mutated group showed higher survival probabilities as time progressed.

To further validate the scientific significance of MGPPI, we collected three cancer patient datasets (breast cancer, bladder cancer, and colorectal cancer) and analyzed them individually. We selected protein-protein interaction samples related to cancer from the HPRD dataset based on proteins present in the patients’ bodies. We successfully predicted the critical binding sites of these proteins using the MGPPI model. Subsequently, based on the incidence of mutations at these protein binding sites within the cancer patient dataset, we categorized each individual into one of two groups: patients with mutated binding sites and patients with non-mutated binding sites. We then analyzed the impact of these mutation sites on the survival time of patients in each cancer group. Finally, Kaplan-Meier curves were generated by analyzing the survival time and status of patients with each type of cancer, incorporating patient grouping information. As show in Figure 7:

From Figure 7, it is evident that there are significant differences in survival probabilities between the two patient groups for each type of disease, indicating the importance of these key sites for human survival. For example, in breast cancer patients, the occurrence of specific mutations at critical amino acid residues of certain proteins may suggest the presence of abnormalities or functional alterations in their bodies. As a result, their resistance against cancer could be weakened, rendering them more vulnerable to its effects and leading to a rapid decline in survival rates over time. Conversely, non-mutated patients at key sites demonstrate higher survival probabilities, indicating a potential survival advantage associated with those specific protein sites. However, in the case of bladder cancer and colorectal cancer patients, the Kaplan-Meier curve results are opposite to those of breast cancer patients, with the mutated groups showing higher survival probabilities over time. This indicates that mutations at certain amino acid residues may not necessarily be harmful to patients, and in some cases, they can even have a positive impact on the treatment of certain cancers, thereby increasing patients’ survival probabilities.

In conclusion, MGPPI accurately predicts crucial amino acid sites in cancer patients that play a significant role in disease resistance, further validating the reliability and scientific soundness of the MGPPI model.

### 3.6 Limitations and future direction

Although MGPPI has demonstrated advantages in protein-protein interaction prediction and binding site prediction, there are still limitations in this study. Firstly, the output of the MGPPI model is a probability value that requires setting a threshold to convert probabilities into classifications. Choosing an inappropriate threshold may result in the model missing some true positive samples, leading to lower evaluation metrics than the actual values.

In future work, we will incorporate the 3D coordinate information of amino acids to predict binding sites during protein-protein interactions. In real-world scenarios, proteins exhibit diverse shapes, and certain amino acids may be located inside the protein due to protein folding or distortion. The likelihood of these amino acids interacting with other proteins is low. Therefore, when discussing PPI and predicting binding sites, it is essential to consider the actual coordinate information of amino acids.

## 4 Conclusion

This paper presents a novel PPI prediction framework called MGPPI based on chemical intuition. MGPPI utilizes MGCN, which consists of 15 graph convolutional layers, to capture the multiscale structure of proteins. It also employs Grad-WAM for visual interpretation. Extensive experiments validate the superiority of this method, demonstrating significant improvements over existing approaches on four human protein datasets and one multi-species dataset. The ability of MGPPI to represent proteins from various species as graph data greatly enhances the model’s generalization capability. Furthermore, MGPPI successfully predicts the interaction between the spike protein of SARS-COV-2 and the human ACE2 receptor protein. By utilizing Grad-WAM, the importance of amino acids is visualized as labels, and the rationality of predicted binding sites is validated based on functional domain and Gene Ontology annotation. Finally, we screened for relevant proteins from samples of three cancer patients and used the MGPPI model to predict the binding sites of these proteins. Based on whether these sites undergo mutations, we divided each type of cancer patient into two groups and investigated the impact of these sites on the survival status of patients with the three types of diseases. The research results indicate that MGPPI enhances the overall generalization and interpretability of PPI prediction models, making it a highly practical tool.

## Data Availability

The data presented in the study are deposited in the Github. accession link: https://github.com/Shiwei-Zhao/MGPPI.
